# 10-Hydroxy-2-decenoic Acid Suppresses Colorectal Cancer Progression by Inhibiting Wnt/β-Catenin Signaling and Promoting Apoptosis

**DOI:** 10.3390/foods15091608

**Published:** 2026-05-06

**Authors:** Yan Lin, Rongjing Cai, Lei Huang, Tianxing Lin, Anqi Lin, Zhenyu Lin, Shoujie Jiang, Yuqi Zhu, Yuan Yuan, Songkun Su

**Affiliations:** 1 College of Bee Science and Biomedicine, Fujian Agriculture and Forestry University, Fuzhou 350002, China; ylin19@qub.ac.uk (Y.L.); 52306009007@fafu.edu.cn (R.C.); leiihuang@fafu.edu.cn (L.H.); tianxinglin0821@fafu.edu.cn (T.L.); 12407044014@fafu.edu.cn (A.L.); 3245602021@stu.fafu.edu.cn (Z.L.); 52507057012@fafu.edu.cn (S.J.); 12507044016@fafu.edu.cn (Y.Z.); 52306009025@fafu.edu.cn (Y.Y.); 2 College of Animal Sciences, Fujian Agriculture and Forestry University, Fuzhou 350002, China; 3 College of Life Sciences, Fujian Agriculture and Forestry University, Fuzhou 350002, China

**Keywords:** colorectal cancer, 10-hydroxy-2-decenoic acid, Wnt/β-catenin pathway, apoptosis, royal jelly

## Abstract

Colorectal cancer (CRC) remains a leading cause of cancer-related death worldwide, and advanced disease continues to show poor prognosis due to therapeutic limitations and drug resistance. Royal jelly (RJ), a natural functional food and dietary supplement, contains 10-hydroxy-2-decenoic acid (10-HDA), a bioactive fatty acid unique to RJ with demonstrated anticancer potential. This study evaluated the anti-CRC effects and underlying mechanisms of 10-HDA through cellular, animal, and transcriptomic approaches. 10-HDA markedly suppressed CRC cell viability with IC_50_ of 2.07 mM and 3.49 mM against HCT 116 and HT-29 cells, respectively, reduced gap closure by 29.30%, elevated intracellular reactive oxygen species (ROS), and attenuated xenograft tumor growth dose-dependently. Preliminary safety evaluation suggested that 10-HDA was well tolerated under the tested conditions, with no significant changes in body weight, serum AST, ALT, or ALP levels, or organ histology. Transcriptomic analysis showed significant enrichment of apoptosis and Wnt/β-catenin pathways. Molecular assessments indicated that 10-HDA was associated with alterations in apoptosis-related features, including increased caspase-3 activity, changes in Bcl-2 family proteins, and elevated ROS levels, as well as with modulation of the Wnt/β-catenin signaling pathway. These changes were consistent with enhanced β-catenin degradation and reduced nuclear translocation. It suggests that Wnt/β-catenin may be involved in the anti-CRC effects of 10-HDA. This study mechanistically clarifies the anti-CRC activity of 10-HDA as a natural food-derived bioactive compound, suggesting its therapeutic potential for Wnt/β-catenin dysregulated CRC.

## 1. Introduction

Colorectal cancer (CRC) continues to impose a major and growing health burden worldwide [1]. In the GLOBOCAN 2022 report, CRC was among the most commonly diagnosed cancers and a leading cause of cancer-related death, with an estimated 1.93 million new cases and more than 900,000 deaths globally [2]. Projections further indicate that, by 2030, annual incidence may exceed 2.2 million and deaths may rise above 1.1 million [3]. Despite progress in clinical care, ranging from surgery to systemic chemotherapy and targeted agents directed at VEGF or EGFR, outcomes for metastatic CRC remain dismal, with 5-year survival still below 15% [4]. Moreover, treatment benefit is frequently constrained by substantial toxicity and the emergence of acquired resistance [5,6]. These challenges underscore the need for additional therapeutic options, particularly agents with novel modes of action and improved safety that could complement existing regimens and help address resistance [7,8].

Functional foods, defined as foods that provide health benefits beyond basic nutrition, have gained increasing attention for their potential in cancer prevention and management. These foods contain bioactive compounds that can modulate cellular processes and signaling pathways implicated in carcinogenesis [9]. Representative examples include curcumin from turmeric, resveratrol from grapes, berberine from various plants, quercetin from multiple fruits and vegetables, and sulforaphane from cruciferous vegetables. These food-derived bioactive compounds have demonstrated anticancer activities via multiple mechanisms, such as promoting apoptosis, causing cell-cycle arrest, regulating inflammatory responses, and disrupting oncogenic signaling pathways including Wnt/β-catenin [10,11,12]. The exploration of functional food components as natural preventive and therapeutic agents represents a promising approach in cancer research.

Royal jelly (RJ), a secretion produced by worker honeybees, is widely recognized as a natural functional food with diverse biological activities, including antimicrobial, anti-inflammatory, antioxidant, immunomodulatory, and potential antitumor effects [13]. Among the various bioactive constituents of RJ, 10-hydroxy-2-decenoic acid (10-HDA) is a characteristic unsaturated medium-chain hydroxy fatty acid found almost exclusively in RJ. Thus, RJ represents the principal natural dietary source of 10-HDA [14,15]. Beyond its role as a quality marker for RJ, 10-HDA itself has attracted growing research interest due to its broad pharmacological properties and emerging anticancer potential [16]. Specifically, 10-HDA (also termed queen bee acid/royal jelly acid) accounts for approximately 1.4–2% of the wet weight of RJ and is widely used as a hallmark indicator of RJ freshness and quality [17]. It has been reported to display multiple pharmacological actions, including antimicrobial, neuroprotective, anti-osteoporotic, hypoglycemic, and anti-inflammatory effects. Importantly, accumulating evidence supports an antitumor efficacy of 10-HDA against diverse cancer models, highlighting it as a promising natural candidate for oncology research [18]. Prior work showed that 10-HDA suppressed lung cancer cell growth and motility by provoking cell cycle arrest and mitochondria-dependent apoptosis, partly through the modulation of ROS-mediated MAPK, STAT3 and NF-κB signaling [19]. In highly aggressive breast cancer cell lines [20], 10-HDA reduced migration, invasion, and angiogenesis by inhibiting the FAK/PI3K/RAC1/ERK cascade and impairing focal adhesion formation [21,22]. In Ehrlich solid tumor-bearing mice, oral administration of 10-HDA, particularly in combination with cyclophosphamide, markedly decreased tumor volume, lowered tumor markers (AFP and CEA), enhanced antioxidant enzyme levels, and promoted apoptosis. These studies suggest that 10-HDA exerts broad antitumor activity through multiple complementary mechanisms [23]. In CRC, recent studies have highlighted the therapeutic potential of 10-HDA. It was demonstrated that 10-HDA exhibited anti-metastatic effects on human colorectal carcinoma HCT 116 and SW480 cells by modulating epithelial–mesenchymal transition (EMT) markers and suppressing pro-migratory/pro-invasive factors [24]. Another study in SW620 human colon cancer cells found that 10-HDA inhibited cell proliferation and migration by promoting apoptosis and oxidative stress [25]. In human colon carcinoma HT-29 and SW 837 cells, 10-HDA modulated apoptosis- and cell cycle-related proteins by upregulating BAX, cleaved forms of caspase-3, caspase-7, caspase-8, caspase-9, and p53, while downregulating Bcl-2 and cyclin D1 [26]. These findings suggest that 10-HDA may inhibit CRC progression by restraining proliferation, invasion and metastasis, as well as triggering cancer cell death through multiple mechanisms.

Given the substantial burden of CRC, the limitations of current therapies, and the encouraging yet incompletely defined anticancer activity of 10-HDA, further mechanistic investigation of this natural compound in CRC models is warranted. In the present study, we systematically evaluated the anti-CRC effects of 10-HDA in vitro and in vivo, and subsequently explored the underlying mechanisms through transcriptomic analysis followed by experimental validation. It provides new mechanistic insights into the therapeutic potential of 10-HDA for CRC and supports its possible application as an adjunct to standard treatment to improve efficacy and/or mitigate drug resistance.

## 2. Materials and Methods

### 2.1. Cell Culture

Human colorectal carcinoma cell lines HCT 116 (ATCC; CCL-247) and HT-29 (ATCC; HTB-38) were purchased from Zhong Qiao Xin Zhou Biotechnology Co., Ltd. (Shanghai, China). Routine culturing was performed using high-glucose Dulbecco’s Modified Eagle Medium (DMEM; Gibco, Carlsbad, CA, USA) supplemented with 10% fetal bovine serum (FBS; ExCell, Beijing, China) and 1% penicillin-streptomycin (Gibco, Carlsbad, CA, USA). Cells were maintained in a 37 °C incubator supplied with 5% CO_2_.

### 2.2. MTT Assay

To assess cell viability, an MTT assay was performed using 10-HDA (purity ≥ 98%; MedChemExpress, Monmouth Junction, NJ, USA), as previously described [27]. Briefly, HCT 116 and HT-29 cells were seeded into 96-well plates at densities of 7 × 10^3^ and 5 × 10^3^ cells/well, respectively, and allowed to adhere overnight. Subsequently, the supernatant was replaced with fresh media containing various doses of 10-HDA (1–5 mM), and cultures were maintained for 48 h. At the end of the treatment, 10 μL of MTT (5 mg/mL; Solarbio, Beijing, China) was added to each well for a 4 h incubation. After aspirating the medium, the formed formazan crystals were dissolved in DMSO (Solarbio, Beijing, China). Optical density (OD) was recorded at 550 nm via a microplate reader (Thermo Fisher Scientific, Waltham, MA, USA). Cell viability was expressed as a percentage of the control, with IC_50_ values calculated using non-linear regression.

### 2.3. Colony Formation Assay

Cells were seeded at a density of 500 cells/well into 6-well plates, and treated with 10-HDA (1–2 mM) for 14 days, with replacement of the 10-HDA-containing medium every two days. Subsequently, cells were fixed by 4% paraformaldehyde (Servicebio, Wuhan, China), followed by staining with 0.1% crystal violet (Beyotime, Shanghai, China), three washes with PBS (Servicebio, Wuhan, China) and photography to count the formed colonies.

### 2.4. Scratch Assay

The effect of 10-HDA on gap closure in HCT 116 cells was evaluated using a scratch assay as previously described [28]. Briefly, HCT 116 cells were plated at a density of 8 × 10^5^ cells/well into 24-well plates equipped with Culture Inserts (Ibidi, Gräfelfing, Germany) and cultured for 24 h to achieve complete confluency. Removal of the inserts created a standardized cell-free gap (approximately 500 μm in width). Subsequently, the monolayers were incubated with either vehicle control or 10-HDA (1–2 mM) for 24 h. The extent of gap closure was visualized and captured using a phase-contrast microscope (Olympus, Tokyo, Japan) at 0, 12, and 24 h post-treatment. For quantitative analysis, the gap area from three random fields was measured using ImageJ software (v1.53k; NIH, Bethesda, MD, USA). The gap closure rate was expressed as the percentage of gap closure, calculated based on the reduction in the gap area at specified time points relative to the initial area (0 h).

### 2.5. Quantification of Intracellular ROS Levels

To monitor ROS levels, HCT 116 cells (5 × 10^5^ cells/well) were cultured in 6-well plates and treated with various concentrations of 10-HDA (0–2 mM) for 3 h. Subsequently, the cells were harvested and treated with 10 μM 2′,7′-dichlorodihydrofluorescein diacetate (DCFH-DA; Beyotime, Shanghai, China) for 30 min at 37 °C in the dark. The cells were then washed with PBS and analyzed via flow cytometry using a BD Accuri C6 Plus flow cytometer (BD, San Jose, CA, USA). DCF fluorescence was captured at excitation and emission wavelengths of 488 nm and 525 nm, respectively. FlowJo software (v10.8.1; FlowJo LLC, Ashland, OR, USA) was utilized to calculate the Mean Fluorescence Intensity (MFI).

### 2.6. In Vivo Anticancer Assays

Experimental protocols were conducted in full compliance with the ARRIVE guidelines [29] and the Guidelines for the Care and Use of Medical Laboratory Animals (Ministry of Health, Beijing, China, 1998). Ethical clearance was granted by the Ethical Review Board of Fujian Agriculture and Forestry University (No. PZCASFAFU24125; issued 25 July 2024). A total of 18 male BALB/c nude mice (7 weeks, 19 ± 2 g) were purchased from Wu’s Laboratory Animal (Fuzhou, China), and housed in a specific pathogen-free (SPF) environment controlled at 22 ± 2 °C and 55 ± 10% humidity, under a 12 h light/dark cycle. Food and water were provided ad libitum. After adapting to the facility for one week, the mice were randomly allocated into four groups (*n* = 6). The sample size was calculated using the resource equation approach [30,31,32]. A prophylactic-therapeutic dosing regimen was adopted. Mice received daily oral gavage of 10-HDA (dissolved in saline) at doses of 100 mg/kg or 200 mg/kg, or vehicle (saline) for 30 consecutive days prior to tumor inoculation. Subsequently, a xenograft model was established by subcutaneously injecting HCT 116 cells (1 × 10^7^) into the flank, as previously described [33]. Daily oral administration continued for an additional 21 days post-inoculation. Tumor dimensions were monitored from day 6 to day 21 post-implantation, and volumes were calculated using the formula V = 0.5 × L × W^2^, where L = length, W = width. Upon reaching the experimental endpoint (control tumors ≈ 800 mm^3^), mice were humanely euthanized via CO_2_ inhalation followed by cervical dislocation, and tumor tissues were harvested for downstream analysis.

### 2.7. Evaluation of In Vivo Biosafety

To assess potential systemic toxicity, blood samples were harvested from healthy and tumor-bearing mice at the experimental endpoint (day 52 post-treatment of 10-HDA). Samples were allowed to clot overnight at 4 °C, followed by centrifugation (3000× *g*, 15 min, 4 °C) to isolate serum. Hepatic function indicators, including aspartate aminotransferase (AST), alanine aminotransferase (ALT), and alkaline phosphatase (ALP), were quantified using commercial enzymatic assay kits (Nanjing Jiancheng Bioengineering Institute, Nanjing, China) in accordance with the manufacturer’s instructions. Furthermore, major organs (heart, liver, spleen, lung and kidney) were excised, fixed, and subjected to hematoxylin and eosin (H&E) staining for histopathological examination.

### 2.8. Transcriptomic Analysis

At the experimental endpoint, xenograft tumor tissues were harvested from the vehicle control and 10-HDA (200 mg/kg)-treated groups. RNA sequencing was performed on a DNBSEQ-T7 platform by Igenebook Biotech-Technology Co., Ltd. (Wuhan, China), which generated 150 bp paired-end reads with an average insert size of 250 bp. Raw reads were processed to remove adapter sequences and discard low-quality reads using Cutadapt (v1.11). The resulting clean reads were aligned to the human reference genome (GRCh38) using HISAT2 (v2.1.0). Gene abundance was quantified via FeatureCounts (v1.6.0) and normalized to FPKM, followed by functional annotation against the NCBI NR database. For differential expression analysis, differentially expressed genes (DEGs) were identified using edgeR with significant thresholds set at FDR < 0.05 and |log2Fold Change| > 1. Gene Ontology (GO) and Kyoto Encyclopedia of Genes and Genomes (KEGG) pathway enrichment analyses were subsequently performed using the clusterProfiler R package (v4.2.2), employing a hypergeometric test (*p* < 0.05) to determine significance. Sequencing data were deposited in the NCBI Sequence Read Archive (SRA) under accession number PRJNA1414200.

### 2.9. RT-qPCR Assays

Total RNA was extracted from HCT 116 cells (2 × 10^5^) or xenograft tumor tissues (10 mg) using TRIzol (SigmaAldrich, St. Louis, MO, USA). After quantification, 1 μg of RNA was reverse-transcribed into cDNA using a reverse transcription kit following the manufacturer’s instructions. qPCR was performed with ChamQ Universal SYBR qPCR Master Mix (Vazyme, Nanjing, China) on a QuantStudioTM 5 System (Thermo Fisher Scientific, Waltham, MA, USA). Primer sequences are provided in Supplementary Table S1.

### 2.10. Western Blotting Assays

Total protein was extracted from HCT 116 cells (2 × 10^5^) or xenograft tumor tissues (10 mg) using RIPA lysis buffer (EpiZyme, Shanghai, China) supplemented with a cocktail of protease and phosphatase inhibitors (Beyotime, Shanghai, China). Following centrifugation, protein samples were resolved by 10% SDS-PAGE and electro-transferred onto polyvinylidene difluoride (PVDF) membranes. The membranes were blocked and subsequently probed overnight at 4 °C with specific primary antibodies targeting GSK3β, p-GSK3β, β-catenin, Bcl-2, BAX, caspase-3, cleaved caspase-3 (all diluted 1:2000; ProteinTech, Wuhan, China). β-actin, lamin B1 and β-tubulin (all diluted 1:20,000; Abclonal, Wuhan, China) served as the internal loading control. After washing, the membranes were incubated with HRP-conjugated goat anti-rabbit IgG (1:20,000; Abclonal, Wuhan, China) for 45 min at room temperature. Protein bands were detected with an enhanced chemiluminescence (ECL) kit (EpiZyme, Shanghai, China) and imaged using the Tanon-6000+ system (TanonImage, Tanon, Shanghai, China).

### 2.11. Hematoxylin and Eosin (H&E) Staining

For histological analysis, 0.5 g of tumor tissue was harvested from the xenograft models and fixed with 4% paraformaldehyde. The samples were subsequently processed for paraffin embedding and sliced into sections of standard thickness. After staining with hematoxylin and eosin (H&E) according to established procedures [34], the slides were scanned and imaged utilizing an Aperio CT6 system (Leica Biosystems, Vista, CA, USA).

### 2.12. Immunohistochemical (IHC) Analysis

Paraffin-embedded tumor sections underwent standard deparaffinization and rehydration. Endogenous peroxidase activity was inactivated by exposure to hydrogen peroxide, followed by blocking of non-specific binding sites using 3% bovine serum albumin (BSA; Solarbio, Beijing, China). Subsequently, sections were subjected to overnight probing at 4 °C with specific primary antibodies targeting Bcl-2 (1:400), BAX (1:200), Ki-67 (1:150), and β-catenin (1:600; all from ProteinTech, Wuhan, China). Detection was achieved via incubation with an HRP-conjugated goat anti-rabbit IgG secondary antibody (1:200; Abclonal, Wuhan, China). Antigen–antibody complexes were visualized using 3,3′-diaminobenzidine (DAB; Beyotime, Shanghai, China) as the chromogen, and nuclei were counterstained with hematoxylin.

### 2.13. Statistical Analysis

Statistical analyses were performed using GraphPad Prism 9.0 (GraphPad Software, San Diego, CA, USA). Group comparisons were evaluated via one-way ANOVA supplemented by Tukey’s post hoc test. Statistical significance was defined as *p* < 0.05. Results are expressed as mean ± standard deviation (SD).

## 3. Results

### 3.1. 10-HDA Inhibited the Proliferation, Gap Closure and ROS Generation of Colorectal Carcinoma Cells

**Figure 1:**
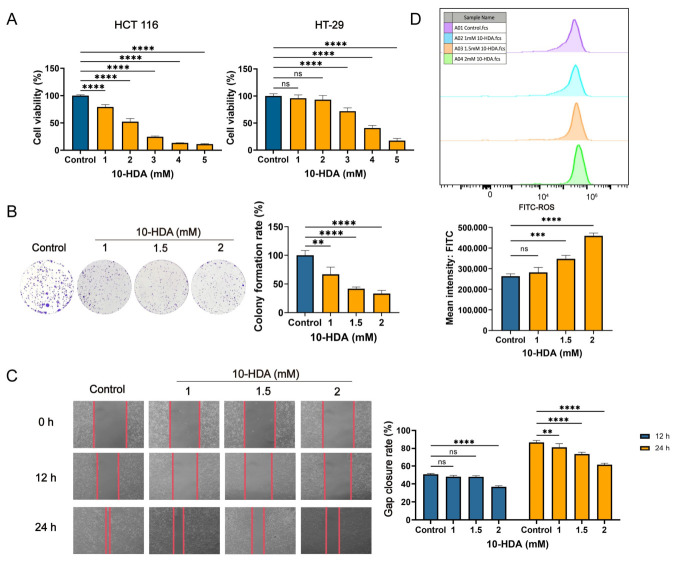
10-HDA inhibited CRC cell growth and gap closure and increased intracellular ROS. (**A**) MTT analysis of HCT 116 and HT-29 cell viability after 48 h exposure to 10-HDA. (**B**) Colony formation of HCT 116 cells following 14 d treatment with 10-HDA. (**C**) Scratch assay analysis of HCT 116 gap closure after 24 h incubation with 10-HDA. (**D**) Flow cytometric analysis of HCT 116 intracellular ROS levels following 3 h exposure to 10-HDA. ** *p* < 0.01, *** *p* < 0.001, **** *p* < 0.0001; ns, no statistical significance. Control, vehicle-treated cells.

As shown in the MTT assay, 10-HDA significantly reduced the viability of HCT 116 and HT-29 cells in a dose-dependent manner, with IC_50_ values of 2.07 ± 0.15 mM and 3.49 ± 0.17 mM, respectively (Figure 1A). Consistently, the colony formation assay confirmed the antiproliferative activity of 10-HDA, decreasing colony numbers in HCT 116 cells by 33.12–66.87% at 1–2 mM (Figure 1B). In the scratch assay, 10-HDA markedly inhibited gap closure in HCT 116 cell monolayers, with a maximal reduction of 29.30%; the effect was stronger at 24 h than at 12 h, indicating time- and dose-dependent suppression of gap closure (Figure 1C). Flow cytometric analysis further showed that a 3 h exposure to 10-HDA increased the HCT 116 intracellular ROS levels in a dose-dependent manner. Compared with the control, ROS was elevated by 1.32-fold and 1.74-fold at 1.5 mM and 2 mM 10-HDA, respectively (Figure 1D).

### 3.2. 10-HDA Inhibited the Growth of Subcutaneous Ectopic CRC Xenografts

**Figure 2:**
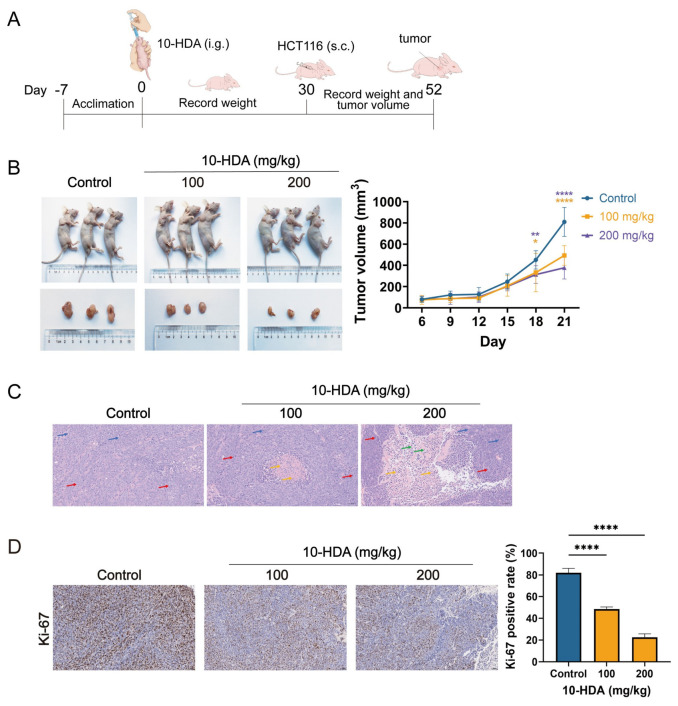
10-HDA inhibited tumor growth and modified the histopathological features of HCT 116 xenografts in vivo. (**A**) Schematic overview of HCT 116 xenograft establishment and 10-HDA administration. (**B**) Representative images of xenograft tumors from saline-treated control mice and 10-HDA-treated mice, with corresponding tumor volume quantification. Control, blue; 100 mg/kg 10-HDA, orange; 200 mg/kg 10-HDA, purple. (**C**) H&E-stained sections of xenograft tumors. Red arrows indicate tumor cells; yellow arrows denote necrotic regions; blue arrows indicate atypical (pathological) mitotic figures; and green arrows indicate inflammatory cell infiltration. (**D**) Ki-67 immunohistochemical staining of xenograft tumors from control and 10-HDA-treated mice. *n* = 6; * *p* < 0.05, ** *p* < 0.01, **** *p* < 0.0001; ns, no statistical significance. Control, saline-treated tumor-bearing mice.

To evaluate the in vivo anti-CRC efficacy of 10-HDA, mice received intragastric 10-HDA for 30 d before tumor implantation, and dosing was maintained after ectopic xenografts were established (Figure 2A). By day 21 post-inoculation, tumor volumes in the 100 and 200 mg/kg groups were decreased by 39.02% and 53.30%, respectively, relative to the saline-treated controls (Figure 2B).

Histopathological evaluation of H&E-stained xenograft sections further highlighted treatment-associated changes in tumor morphology. Control tumors consisted of densely packed cells with marked atypia, coarse chromatin, and frequent mitotic figures. Tumors from the 100 mg/kg group largely resembled controls in overall architecture and mitotic activity, yet showed scattered necrotic foci with nuclear fragmentation and cellular debris. The 200 mg/kg group exhibited more pronounced necrosis, with broad confluent areas containing abundant karyorrhectic material and cellular remnants, accompanied by mild inflammatory cell infiltration. These findings supported the tumor growth-inhibitory effect of 10-HDA in vivo (Figure 2C).

Consistently, immunohistochemistry (IHC) showed a significant reduction in Ki-67 positive cells in the 10-HDA-treated tumors, suggesting suppressed proliferative activity (Figure 2D).

### 3.3. Preliminary Safety Assessment of 10-HDA

**Figure 3:**
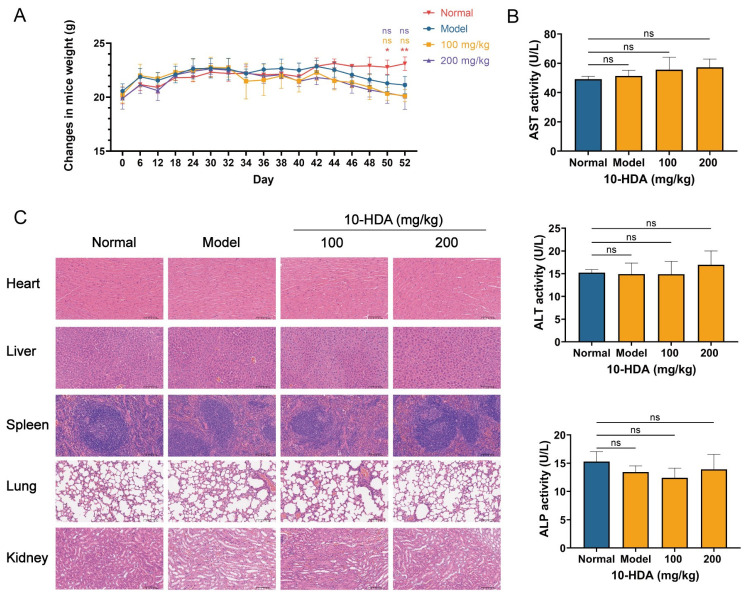
Evaluation of the safety profile of 10-HDA treatment. (**A**) Mice weight changes compared with normal mice. Normal, red; model, blue; 100 mg/kg 10-HDA, orange; 200 mg/kg 10-HDA, purple. (**B**) Serum levels of liver function markers (AST, ALT, and ALP). (**C**) Representative H&E-stained histopathological sections of the heart, liver, spleen, lung and kidney. All analyses were performed on samples from healthy mice (normal control), and saline (model)- and 10-HDA-treated mice bearing HCT 116 xenograft tumors. *n* = 6; * *p* < 0.05, ** *p* < 0.01; ns, no statistical significance.

Throughout the experimental period, the weight of healthy mice gradually increased, which was significantly higher than that of the tumor-bearing ones at the experimental endpoint. However, there was no significant difference between 10-HDA- and saline-treated tumor-bearing mice, indicating that 10-HDA did not induce severe body weight loss and was generally tolerated (Figure 3A).

Serum liver function indices, including AST, ALT, and ALP, were comparable to those of healthy mice, suggesting no detectable 10-HDA-associated hepatic injury under the current dosage (Figure 3B).

Compared with healthy mice, H&E staining of the heart, liver, spleen, lung and kidney from 10-HDA-treated mice showed well-preserved tissue architecture. No obvious inflammatory cell infiltration, hemorrhage, necrosis, or fibrosis was detected in any of the examined organs. All tissues retained normal histological features, including orderly myocardial fiber organization, intact hepatic lobular structure, clear separation of splenic white and red pulp, preserved alveolar morphology, and well-defined renal glomeruli and tubules (Figure 3C).

### 3.4. Transcriptomic Profiling Revealed 10-HDA-Mediated Gene Expression Changes in Ectopic Xenograft Tumors

**Figure 4:**
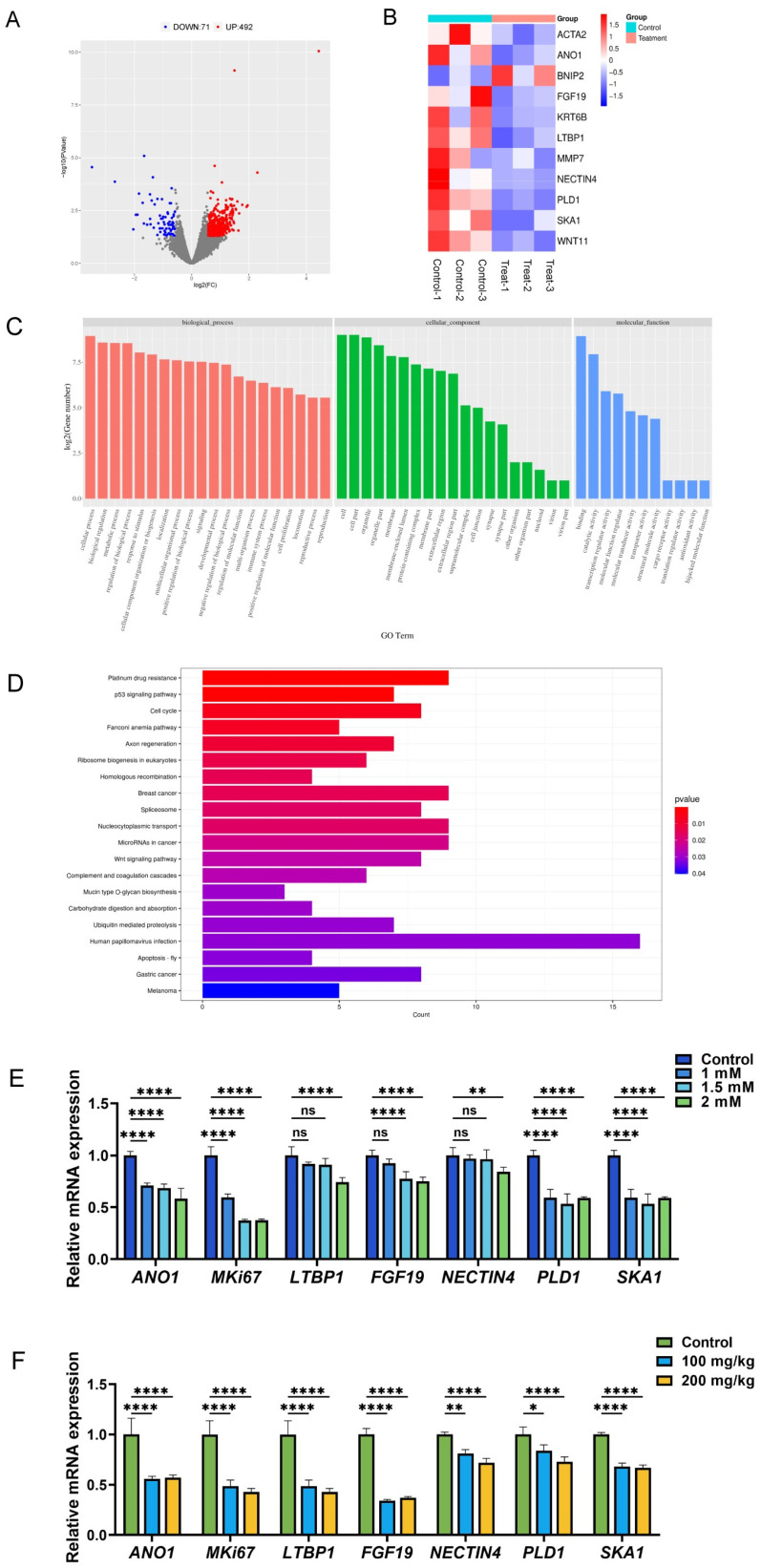
Transcriptomic analysis of ectopic xenograft tumor tissues derived from HCT 116 cells in 10-HDA- and saline-treated (control) mice. (**A**) Volcano plot of DEGs. Each dot represents one gene; red indicates significantly upregulated genes and green indicates significantly downregulated genes (FDR < 0.05 and |log2FoldChange| > 1). (**B**) Heatmap of representative DEGs related to tumor progression. Colors indicate relative expression (red, upregulation; blue, downregulation). (**C**) GO enrichment of DEGs showing top terms in biological process (BP, red), cellular component (CC, green), and molecular function (MF, blue). (**D**) Top 20 enriched KEGG pathways of DEGs. The x-axis shows the number of DEGs in each pathway; bar color represents statistical significance (adjusted *p*-value). (**E**) Relative mRNA expression levels of *ANO1*, *MKi67*, *LTBP1*, *FGF19*, *NECTIN4*, *PLD1*, and *SKA1* in HCT 116 cells treated with 10-HDA or vehicle (control). Control, dark blue; 1.0 mM 10-HDA, blue; 1.5 mM 10-HDA, light blue; 2.0 mM 10-HDA, green. (**F**) Relative mRNA expression levels of *ANO1*, *MKi67*, *LTBP1*, *FGF19*, *NECTIN4*, *PLD1*, and *SKA1* in xenograft tumors from 10-HDA- or saline-treated (control) mice. Control, green; 100 mg/kg 10-HDA, blue; 200 mg/kg 10-HDA, yellow. * *p* < 0.05, ** *p* < 0.01, **** *p* < 0.0001; ns, no statistical significance.

To explore how 10-HDA modulates the transcriptomic profiles of ectopic xenograft tumors, tumor tissues from the mice treated with saline or 10-HDA (200 mg/kg) were collected for RNA sequencing (three biological replicates per group, six libraries in total). As shown in Figure 4A, 563 differentially expressed genes (DEGs) were detected in the tumors from the 10-HDA group relative to saline controls, including 492 upregulated and 71 downregulated genes (Table S2).

To gain insight into the functional relevance of these DEGs, we examined representative genes related to tumor progression (Figure 4B). 10-HDA markedly increased the expression of the pro-apoptotic gene *BNIP2*, whereas genes implicated in proliferation and migration (e.g., *ACTA2*, *ANO1*) were significantly reduced. Notably, *WNT11*, encoding a ligand of the Wnt pathway, was also suppressed in the 10-HDA-treated tumors. GO analysis showed that DEGs were significantly enriched in pathways associated with cellular and metabolic regulation and cell proliferation (Figure 4C and Table S3). KEGG enrichment further highlighted pathways associated with apoptosis and Wnt signaling (Figure 4D and Table S4). To further validate the transcriptomic findings, the mRNA expression of several key downregulated DEGs related to tumor proliferation and progression, including *ANO1*, *MKi67*, *LTBP1*, *FGF19*, *NECTIN4*, *PLD1*, and *SKA1*, was assessed by qRT-PCR. In agreement with the RNA-seq data, 10-HDA significantly reduced the expression of these genes in a dose-dependent manner in vitro (Figure 4E), and similar effects were observed in xenograft tumor tissues in vivo (Figure 4F). These results suggested that 10-HDA may exert antitumor effects at least in part by suppressing tumor proliferation, inhibiting Wnt signaling and promoting apoptosis.

### 3.5. 10-HDA Induced Apoptosis in HCT 116 Cells and Subcutaneous HCT 116 Xenograft Tumors

**Figure 5:**
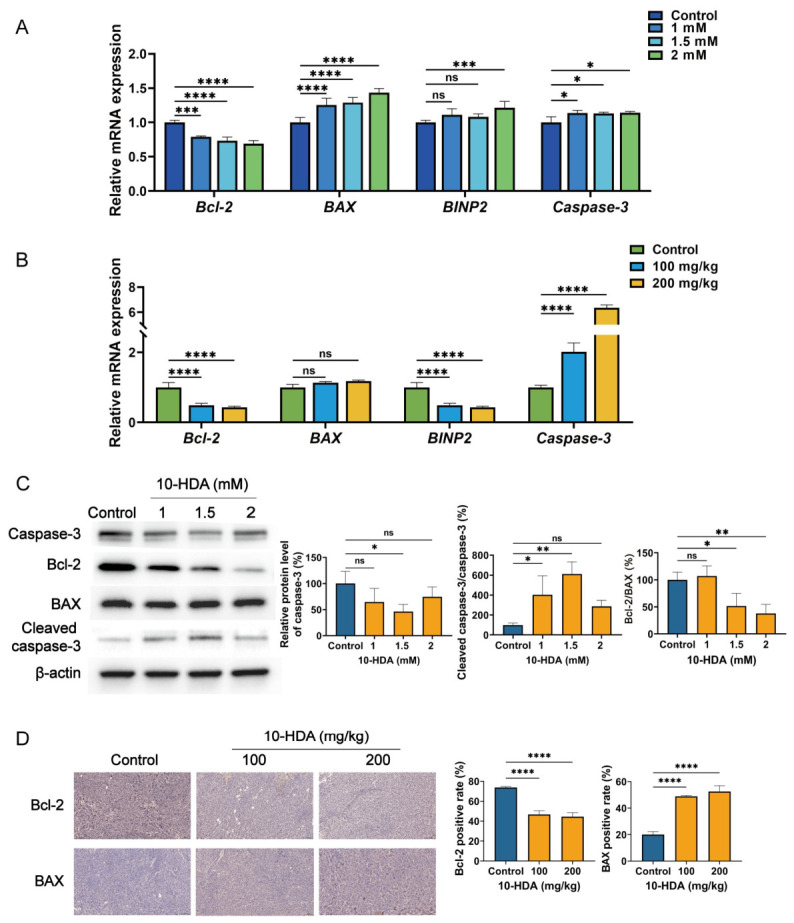
10-HDA induced apoptosis in HCT 116 cells and subcutaneous HCT 116 xenograft tumors. (**A**) Relative mRNA expression levels of *Bcl-2*, *BAX*, *BINP2*, and *caspase-3* in HCT 116 cells treated with 10-HDA or vehicle (control). Control, dark blue, 1.0 mM 10-HDA, blue; 1.5 mM 10-HDA, light blue; 2.0 mM 10-HDA, green. (**B**) Relative mRNA expression levels of *Bcl-2*, *BAX*, *BINP2*, and *caspase-3* in xenograft tumors from 10-HDA- or saline-treated (control) mice. Control, green; 100 mg/kg 10-HDA, blue; 200 mg/kg 10-HDA, yellow. (**C**) Western blotting analysis of caspase-3, Bcl-2, BAX, and cleaved caspase-3 protein levels, as well as the calculated cleaved caspase-3/caspase-3 and Bcl-2/BAX ratios in HCT 116 cells treated with 10-HDA or vehicle (control). (**D**) Immunohistochemical staining of Bcl-2 and BAX in xenograft tumors from 10-HDA- or saline-treated (control) mice. * *p* < 0.05, ** *p* < 0.01, *** *p* < 0.001, **** *p* < 0.0001; ns, no statistical significance.

To further explore the impact of 10-HDA on apoptosis, we measured the mRNA levels of core apoptotic genes. In HCT 116 cells, 10-HDA treatment significantly decreased the mRNA level of the anti-apoptotic gene *Bcl-2*, while upregulating the pro-apoptotic genes *BAX*, *BINP2*, and *caspase-3* (Figure 5A). Similarly, in the subcutaneous xenograft model, 10-HDA administration drastically upregulated *caspase-3* mRNA and downregulated *Bcl-2* mRNA, although *BAX* remained unchanged and *BINP2* was decreased (Figure 5B). Consistent with these transcriptional changes, protein analysis revealed that in HCT 116 cells, 10-HDA caused a significant and dose-dependent reduction in the Bcl-2/BAX protein ratio and promoted the activation of caspase-3, as evidenced by the increased cleaved caspase-3/caspase-3 ratio (Figure 5C). Consistent with these in vitro results, immunohistochemical staining of xenograft tumors showed decreased Bcl-2 expression and increased BAX expression in tissues from the 10-HDA-treated mice compared with controls (Figure 5D).

### 3.6. 10-HDA Affects Wnt/β-Catenin Signaling Pathway Components in HCT 116 Cells and Xenograft Tumors

**Figure 6:**
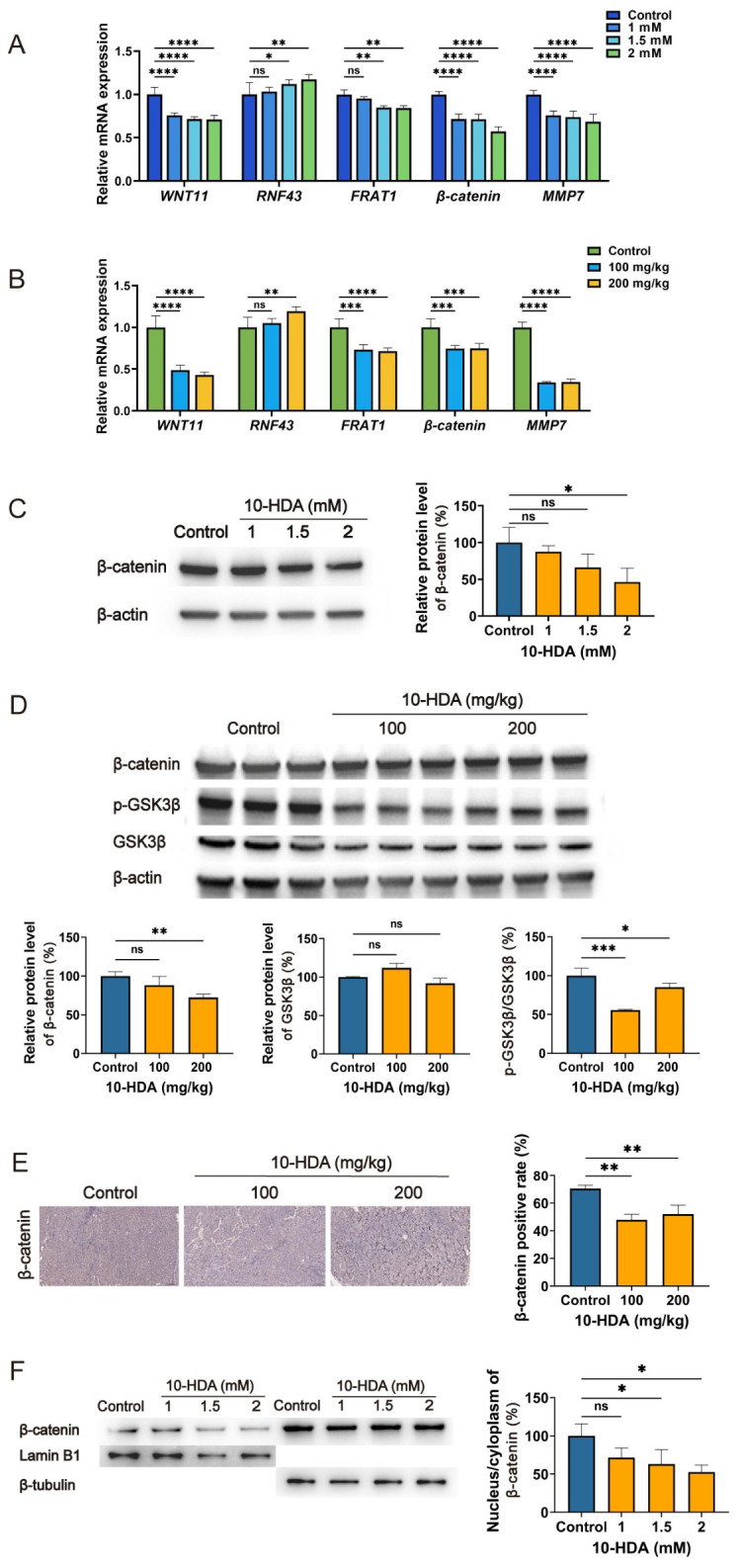
Involvement of Wnt/β-Catenin signaling in the anti-CRC effects of 10-HDA. (**A**) Relative mRNA expression levels of *WNT11*, *RNF43*, *FRAT1*, *β-catenin*, and *MMP7* in HCT 116 cells treated with 10-HDA or vehicle (control). Control, dark blue; 1.0 mM 10-HDA, blue, 1.5 mM 10-HDA, light blue; 2.0 mM 10-HDA, green. (**B**) Relative mRNA expression levels of *WNT11*, *RNF43*, *FRAT1*, *β-catenin*, and *MMP7* in xenograft tumors from 10-HDA- or saline-treated (control) mice. Control, green; 100 mg/kg 10-HDA, blue; 200 mg/kg 10-HDA, yellow. (**C**) Western blotting analysis of β-catenin expression in HCT 116 cells treated with 10-HDA or vehicle (control). (**D**) Western blotting analysis of β-catenin, p-GSK3β and GSK3β in xenograft tumor tissues from 10-HDA- and saline-treated (control) mice. (**E**) Immunohistochemical staining of β-catenin in xenograft tumors. (**F**) Western blotting analysis and densitometric quantification of the nuclear and cytoplasmic β-catenin in HCT 116 cells after 10-HDA or vehicle (control) treatment. * *p* < 0.05, ** *p* < 0.01, *** *p* < 0.001, **** *p* < 0.0001; ns, no statistical significance.

As Wnt/β-catenin signaling pathway is involved in both the initiation and progression of tumors, we investigated the modulatory effectiveness of 10-HDA on the key components of this cascade. qPCR analysis demonstrated that 10-HDA significantly decreased the mRNA levels of *WNT11*, *FRAT1*, *β-catenin*, and *MMP7*, while increasing *RNF43* expression, in both HCT 116 cells and xenograft tumor tissues (Figure 6A,B). In HCT 116 cells, 10-HDA decreased β-catenin protein expression (Figure 6C). Consistent with the in vitro findings, 10-HDA also reduced β-catenin levels in tumor tissues. Notably, the decrease in the p-GSK3β/total GSK3β ratio suggested enhanced GSK3β activity, a change predicted to promote β-catenin phosphorylation, destabilization, and degradation (Figure 6D). Immunohistochemistry further supported these findings by showing weaker β-catenin staining in tumor sections (Figure 6E). Collectively, these data revealed an association between 10-HDA treatment and modulated Wnt/β-catenin signaling components, changes that aligned with the observed anti-tumor activity in CRC.

Since nuclear translocation of β-catenin is essential for Wnt pathway activation, we further examined whether 10-HDA affected β-catenin subcellular distribution using nuclear-cytoplasmic fractionation. Fraction purity was confirmed by β-tubulin and Lamin B1 as markers for the cytoplasmic and nuclear fractions, respectively. Western blotting showed a dose-dependent decline in nuclear β-catenin with increasing 10-HDA concentrations. Densitometric quantification further demonstrated that the nuclear-to-cytoplasmic β-catenin ratio was significantly reduced following treatment with 1.5 mM and 2 mM 10-HDA compared with the control group, whereas the 1 mM treatment showed no significant difference (Figure 6F). These results indicated that 10-HDA inhibited β-catenin nuclear translocation from the cytoplasm, a key step required for Wnt pathway activation, providing a potential mechanism for its anti-tumor activity in CRC.

## 4. Discussion

CRC continues to be a leading cause of cancer mortality globally [1,2]. Despite improved surgery and systemic regimens, metastatic CRC still has a 5-year survival below 15%, with recurrence and treatment-limiting toxicity often accompanying drug resistance [35]. Accumulating evidence indicates that functional foods and their bioactive ingredients hold substantial promise for cancer prevention and management. Nutrients with bioactive properties, such as curcumin, resveratrol, berberine, quercetin, and sulforaphane, can suppress tumor cell growth, motility, and invasiveness through the modulation of redox balance, apoptotic processes, and signaling cascades including Wnt/β-catenin, PI3K/Akt, and NF-κB [36,37,38,39,40]. In CRC, these food-derived bioactives not only act directly on tumor cells, but also influence the tumor-associated microenvironment through regulation of gut microbiota composition, maintenance of intestinal barrier integrity, and preservation of intestinal homeostasis [13]. Consistent with this growing body of evidence, our findings identify 10-HDA, an unsaturated fatty acid derived from RJ, as a bioactive component with significant antitumor activity in CRC [41]. Previous studies have shown that 10-HDA inhibits proliferation and metastasis. A recent study by Jovanović et al. reported that 10-HDA suppressed the migratory and invasive capacity of HCT 116 and SW-480 cells through modulation of epithelial–mesenchymal transition (EMT)-related markers, including E-cadherin, N-cadherin, vimentin, and Snail, providing evidence for its antimigratory and anti-invasive properties in CRC [24]. While that study focused primarily on cell motility and EMT marker regulation, it did not explore the upstream signaling pathways governing tumor cell proliferation and survival, nor did it employ in vivo tumor models or transcriptomic approaches to identify the broader molecular targets of 10-HDA. Consequently, in CRC, the comprehensive signaling basis underlying the observed anti-tumor effects of 10-HDA, including the potential involvement of the Wnt/β-catenin pathway, has not been fully elucidated. Here, we combined in vitro assays, xenograft models, and transcriptomic analysis to characterize the antitumor effects of 10-HDA and explore the potential underlying molecular pathways. The biological effects of 10-HDA may be influenced, at least in part, by its physicochemical properties [42]. Owing to its amphiphilic nature as a medium-chain unsaturated fatty acid containing both hydroxyl and carboxyl groups, 10-HDA may readily interact with membrane phospholipids, thereby facilitating cellular uptake and intracellular distribution. In addition, its α, β-unsaturated carboxylic acid moiety may contribute to redox-related reactivity, which could be associated with the increased ROS levels observed following treatment [43]. Its lipophilic character may also support access to intracellular membrane compartments [44], including mitochondria, and thereby influence apoptotic signaling. These interpretations remain speculative and require direct experimental validation, but they offer a plausible chemical basis for the antitumor activity of 10-HDA.

In the in vitro assays, 10-HDA significantly diminished the CRC cell viability and clonogenic capacity (Figure 1A,B), suggesting a long-term impairment of proliferative potential in tumor cells. Consistent with prior observations in other tumor models [23], the ROS levels in CRC cells rose with the increasing dose of 10-HDA (Figure 1D). In xenografts, dosing of 10-HDA before and after implantation of HCT 116 slowed tumor expansion in a dose-dependent manner (Figure 2). Treated tumors showed larger necrotic regions, reduced atypia, and lower percentage of Ki-67 positive cells, aligning with the anti-proliferative effect in vitro. Tolerability was supported by the stable body weight, unchanged AST/ALT/ALP, and absence of histological injury in major organs (Figure 3), suggesting a favorable therapeutic index [45].

Apoptosis represents a form of programmed cell death, and the capability of evading this process is considered as a defining characteristic of cancer. Hence, recovering the responsiveness of malignant cells to apoptotic signals could be a promising therapeutic strategy for the management of CRC [46,47]. In the intrinsic apoptosis, Bcl-2 protein family, involving anti-apoptotic members such as Bcl-2 and pro-apoptotic members such as BAX, play an important role in governing cell death [48]. In this study, exposure to 10-HDA resulted in a decline in the Bcl-2/BAX protein ratio of HCT 116 cells (Figure 5C), as well as lower Bcl-2 and higher BAX in xenografts (Figure 5D), indicating the engagement of intrinsic death signaling. Consistently, 10-HDA increased the cleaved caspase-3/caspase-3 ratio in HCT 116 cells (Figure 5C), further supporting activation of the intrinsic apoptosis. Transcriptomics revealed that 10-HDA treatment significantly upregulated *BNIP2*, a well-established pro-apoptotic gene that is known to mediate mitochondrial apoptosis through ROS generation and the intrinsic apoptotic pathway [35,49], and regulated genes associated with apoptosis pathway, corroborating the protein-level findings (Figure 4B). The induction of apoptosis likely contributed to the observed reductions in tumor growth and cell proliferation (Figure 1 and Figure 2), suggesting that promoting apoptosis was one of the primary mechanisms through which 10-HDA exerted the anticancer effects [50]. Notably, the histopathological examination revealed extensive necrotic regions in 10-HDA-treated tumors (Figure 2C), which might be partially attributable to apoptotic cell death. Consistent with previous studies that 10-HDA induced apoptosis of lung cancer cells through ROS-mediated pathways, the present findings implied that ROS generation might serve as an upstream signal that triggered the intrinsic apoptotic cascade, as 10-HDA dose-dependently modulated the intracellular ROS levels (Figure 1D) and Bcl-2 family proteins (Figure 5). Hence, it was speculated that 10-HDA could effectively restore the apoptotic potential of CRC cells, thereby suppressing tumor progression.

In many cases of tumorigenesis, Wnt/β-catenin signaling pathway is abnormally activated, driving tumor initiation, progression and metastasis [51,52]. When Wnt ligands are absent, β-catenin in the cytoplasm undergoes phosphorylation mediated by a protein degradation complex composed of GSK3β, APC and Axin, leading to ubiquitination and eventual proteasomal degradation of β-catenin [53]. When Wnt signaling pathway becomes active, phosphorylation of β-catenin is suppressed. It enables β-catenin to build up in the cytoplasm and subsequently move into the nucleus, where it stimulates the expression of genes regulating cellular processes such as proliferation, survival, and stemness [54,55]. In this study, transcriptomic analysis of tumor tissues revealed marked downregulation of key components of Wnt signaling (e.g., *WNT11*) and enrichment of Wnt pathway (Figure 4B,D), suggesting the involvement of Wnt signaling in the anticancer mechanism of 10-HDA. Indeed, Western blotting analysis demonstrated that 10-HDA significantly decreased the p-GSK3β/GSK3β protein ratio (Figure 6D), suggesting enhanced GSK3β activity [56,57]. Such a reduction was accompanied by the significant downregulation of β-catenin protein expression both in vitro in HCT 116 cells and in vivo in tumor tissues (Figure 6C–E). Since GSK3β is an essential element in the β-catenin degradation machinery, it suggests that 10-HDA treatment may facilitate the phosphorylation process of β-catenin, followed by its proteolytic breakdown. This cascade of events ultimately is likely responsible for a decrease in the total abundance of β-catenin protein within the cells [58].

**Figure 7:**
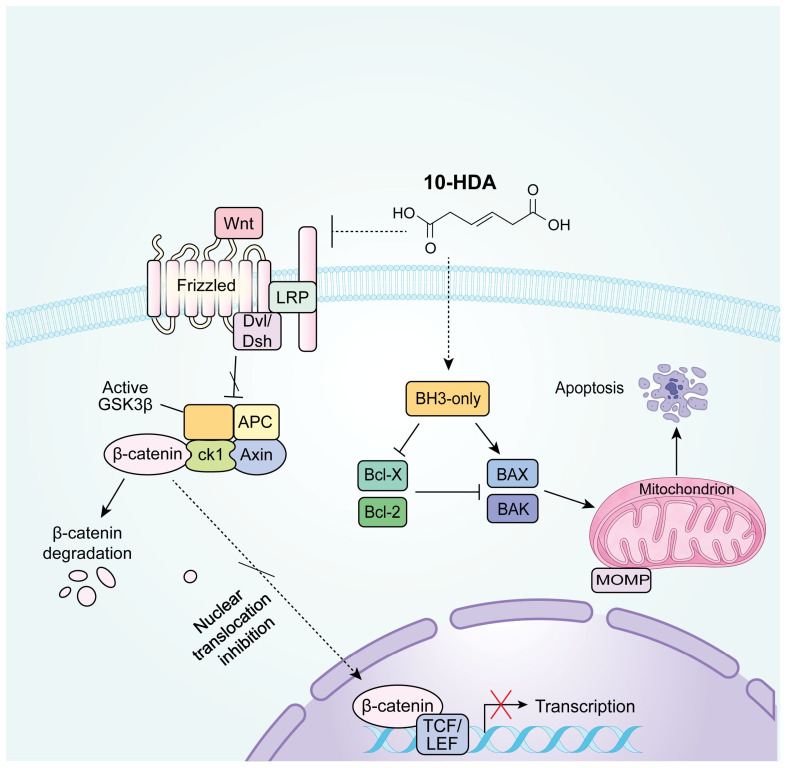
Schematic illustration of the proposed molecular mechanisms by which 10-HDA may exert anticancer effects against CRC, involving the promotion of intrinsic apoptosis and the attenuation of Wnt/β-catenin signaling. Created using Adobe Illustrator 2026 and adapted from Liu et al. (2022) [65] and Huang et al. (2019) [66].

Importantly, the movement of β-catenin from the cytoplasm to the nucleus constitutes an essential stage for Wnt pathway activation. Within the nucleus, β-catenin serves as a transcriptional co-activator that partners with TCF/LEF transcription factors, removing transcriptional suppressors such as Groucho and bringing in various co-regulatory molecules, including enzymes that modify histone structures. It results in the expression of downstream effector genes related to tumorigenesis, such as *c-Myc* and *Cyclin D1* [59,60,61]. Here, nuclear-cytoplasmic fractionation assays demonstrated that 10-HDA gradually decreased the nuclear β-catenin protein levels, with a significant reduction in the nuclear/cytoplasmic ratio at higher concentrations (1.5 mM and 2 mM) (Figure 6). It suggested that the 10-HDA-induced reduction in total β-catenin was accompanied by a decrease in its nuclear accumulation, which may subsequently attenuate the transcriptional activation of Wnt target genes associated with promotion of cell proliferation, inhibition of apoptosis, and enhancement of metastasis [62,63]. The inhibition of β-catenin nuclear translocation is particularly important, as it represents a critical checkpoint in the Wnt signaling and provides a potential mechanistic explanation for the observed reductions in cell proliferation and tumor growth (Figure 1 and Figure 2) [64]. These data suggest that the anticancer effects of 10-HDA are closely associated with the suppression of Wnt/β-catenin signaling. This suppression is potentially driven by the downregulation of β-catenin in the cytoplasm and a concurrent reduction in its nuclear localization, which together modulate the pathway at multiple levels (Figure 7).

10-HDA possesses distinct characteristics that differentiate it from other food-derived anticancer compounds. As the signature bioactive fatty acid unique to RJ, 10-HDA represents a molecular class (medium-chain fatty acid) less explored in the context of functional food anticancer research, compared to the extensively studied polyphenols and alkaloids. Its lack of obvious systemic toxicity, demonstrated by the absence of significant adverse effects in our in vivo experiments, and its presence in RJ, which has an established history of human consumption as a dietary supplement, further support its potential as a food ingredient worthy of further investigation for cancer preventive strategies. The present study provides the first mechanistic characterization of 10-HDA’s anti-CRC activity, highlighting its association with apoptosis induction and the modulation of Wnt/β-catenin signaling. These results indicate that 10-HDA is a functional food-derived molecule candidate for further exploration for its chemopreventive potential.

Although the present study generated positive results, several limitations merit consideration. First, the experiments were primarily conducted using a single CRC cell line (HCT 116) which harbored specific genetic mutations, including a constitutively active β-catenin mutation [67,68]. Validation across genetically diverse CRC models, including microsatellite instability (MSI) and microsatellite stable (MSS) lines, is needed to further evaluate the anticancer spectrum of 10-HDA [69,70]. Thus, the current mechanistic conclusions should be interpreted strictly within the context of this specific model, as the reliance on HCT 116 cells limits the generalizability of our findings until these key observations are validated in additional CRC cell lines. Second, subcutaneous xenograft did not capture the orthotopic microenvironment of CRC, thus orthotopic and metastasis-competent models would better elucidate the effects of 10-HDA on tumor metastasis, immune cell infiltration, and stromal interactions [71,72,73]. Third, the direct target of 10-HDA is still unknown. It can be further investigated by exploring ligand-receptor events, destruction-complex integrity, and rescue experiments using ROS scavengers or β-catenin stabilization approaches [74,75]. While we have provided biochemical and transcriptomic evidence for the modulation of the Wnt/β-catenin pathway and apoptosis, the exact upstream molecular initiation events require further investigation. Specifically, the current study did not include functional assays (e.g., rescue experiments, pharmacological modulation, or pathway-specific reporters) to directly demonstrate causal involvement of the Wnt/β-catenin pathway. Therefore, future studies should incorporate more comprehensive mechanistic experiments, such as targeted pathway modulation or direct validation of downstream targets, to substantiate these correlative observations [76]. Fourth, given that 10-HDA significantly reduces cell viability and proliferation, the observed inhibition of gap closure in scratch assays may be confounded by its anti-proliferative effects. Therefore, further studies employing more appropriate proliferation-blocking controls and migration/invasion-specific assays with minimal proliferation changes are needed to differentiate between its direct anti-migratory effects and its indirect effects via proliferation inhibition [77]. Fifth, although no overt gross toxicity was observed in the xenograft models, our current in vivo toxicity evaluation is preliminary. Comprehensive toxicological and pharmacokinetic profiling, including hematological and serum biochemical analyses, will be imperative in future studies to fully establish the safety profile of 10-HDA [78]. Sixth, while our prophylactic-treatment design offers translational insights into the chemopreventive and adjuvant potential of 10-HDA by pre-conditioning the host environment, standard post-engraftment therapeutic models are required to evaluate its direct anti-tumor efficacy [79]. Finally, longer treatment schedules and combination studies with standard therapies are worth to be conducted to evaluate the durability and safety of 10-HDA, and to test its capability in enhancing the responses of drug-resistant models [80,81].

## 5. Conclusions

This study provides initial evidence that 10-HDA effectively suppresses CRC progression both in vitro and in vivo through intrinsic apoptosis and modulation of Wnt/β-catenin signaling pathway. Specifically, 10-HDA inhibits CRC cell proliferation and promotes apoptosis; meanwhile, it significantly suppresses tumor growth in xenograft mice. These findings suggest an association between 10-HDA’s anti-tumor activity and modulation of Wnt/β-catenin signaling; however, direct functional assays are warranted to establish causality. The absence of significant systemic toxicity in our current in vivo model provides preliminary evidence of tolerability for 10-HDA, warranting further study in CRC. These findings not only enhance current knowledge about the molecular mechanisms underlying the anticancer activity of 10-HDA as a functional food-derived bioactive compound, but also provide a strong rationale for its further investigation as a novel functional ingredient in the context of diet-based cancer preventive and therapeutic strategies.

## Data Availability

The original contributions presented in this study are included in the article/Supplementary Material. Further inquiries can be directed to the corresponding author. The original RNA sequence metadata are openly available in the NCBI Sequence Read Archive (SRA) database with the accession number PRJNA1414200.

## Supplementary Materials

The following supporting information can be downloaded at: https://www.mdpi.com/article/10.3390/foods15091608/s1, Figure S1. Original Western blotting images. Table S1. Primers used for qPCR. Table S2. Differentially expressed genes (DEGs) in the ectopic xenograft tumor tissues from mice treated 10-HDA vs. those treated saline. Table S3. GO enrichment of DEGs. Table S4. KEGG enrichment of DEGs.

## Abbreviations

The following abbreviations are used in this manuscript:

CRC	Colorectal cancer
RJ	Royal jelly
10-HDA	10-Hydroxy-2-decenoic acid
ROS	Reactive oxygen species
H&E	Hematoxylin and eosin
DEG	Differentially expressed gene
GO	Gene Ontology
KEGG	Kyoto Encyclopedial of Genes and Genomes
VEGF	Vascular endothelial growth factor
EGFR	Epidermal growth factor receptor
