# Supplementary material for: 10-Hydroxy-2-decenoic Acid Suppresses Colorectal Cancer Progression by Inhibiting Wnt/β-Catenin Signaling and Promoting Apoptosis
Source: Foods. 2026 May 6;15(9):1608. doi: 10.3390/foods15091608 (PMC13163642; doi:10.3390/foods15091608)

Figure S1. Original Western blotting images

Source-DATA-Figure.5

Fig.5C: Bcl-2 Bax

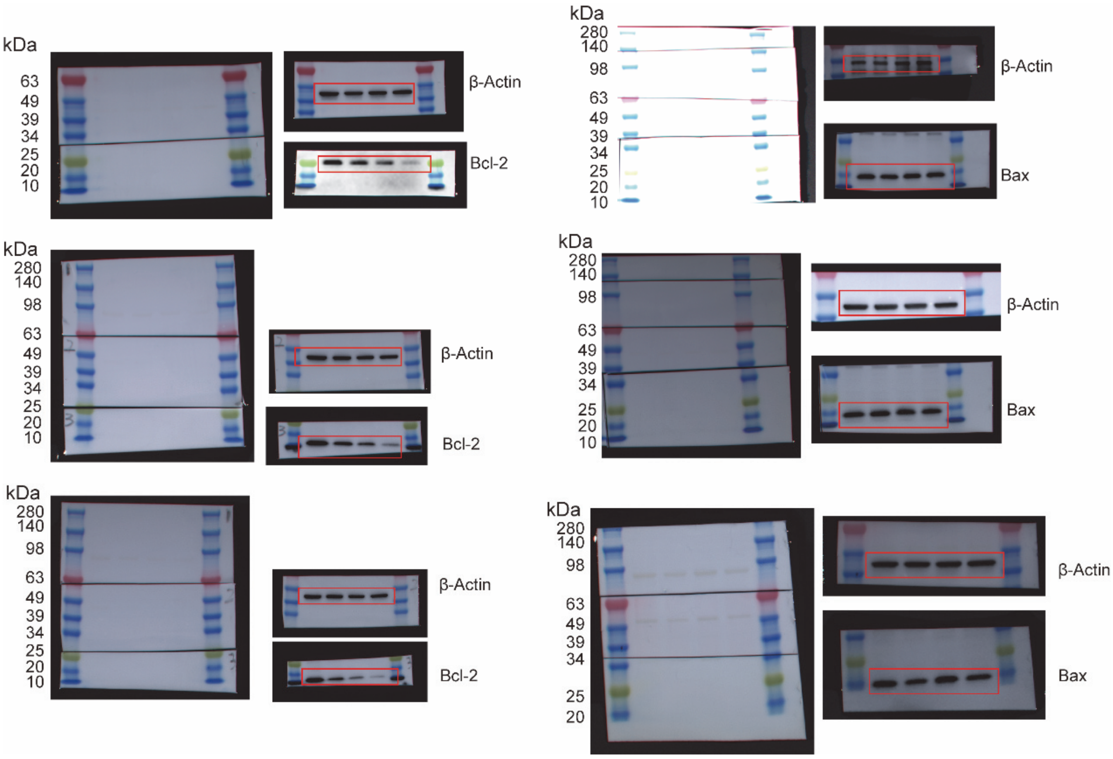

Source-DATA-Figure.5

Fig.5C: cleaved caspase-3 and caspase-3

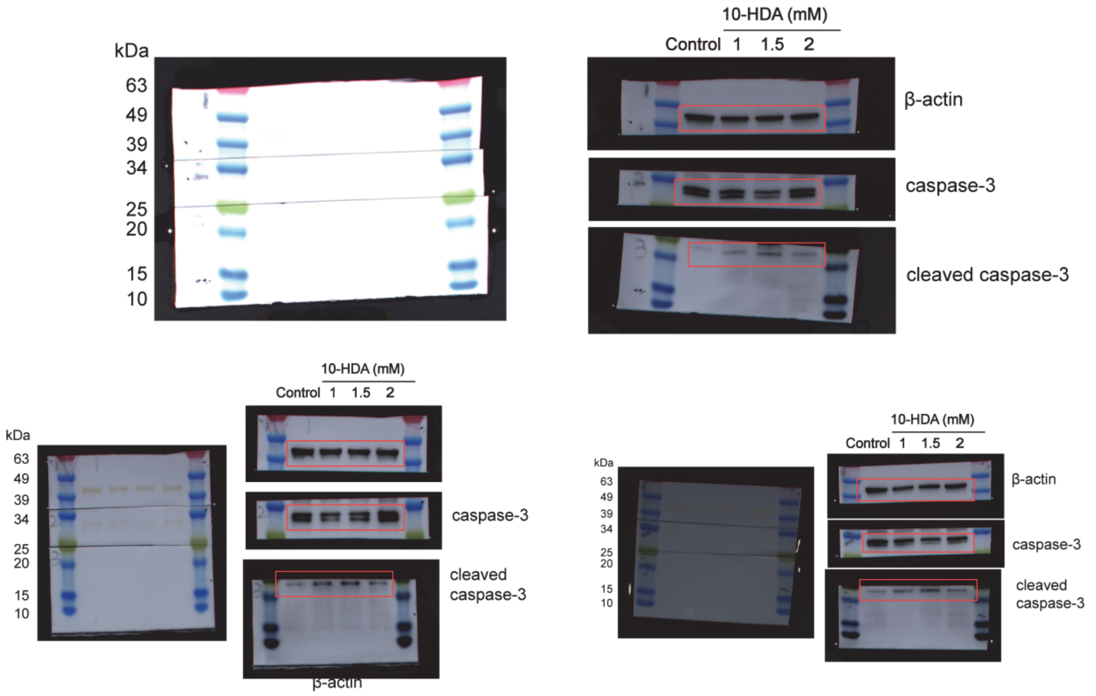

Source-DATA-Figure.6

Fig.6C: β-catenin

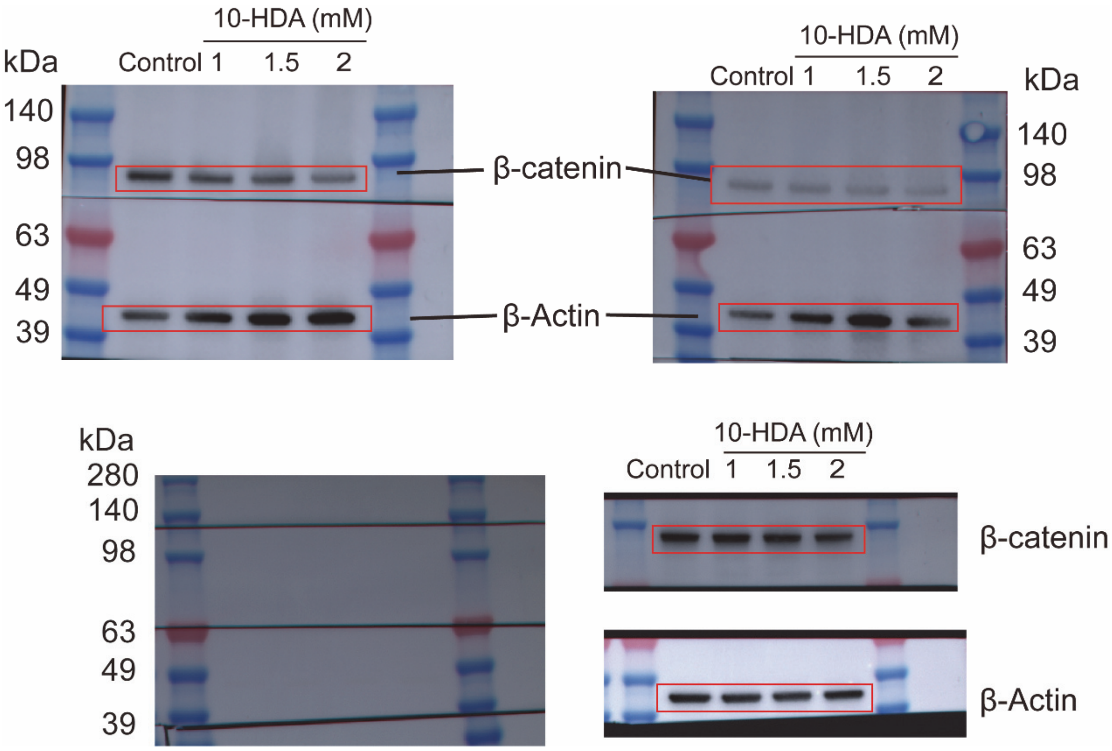

Source-DATA-Figure.6

Fig.6D: β-catenin p-GSK3β GSK3β

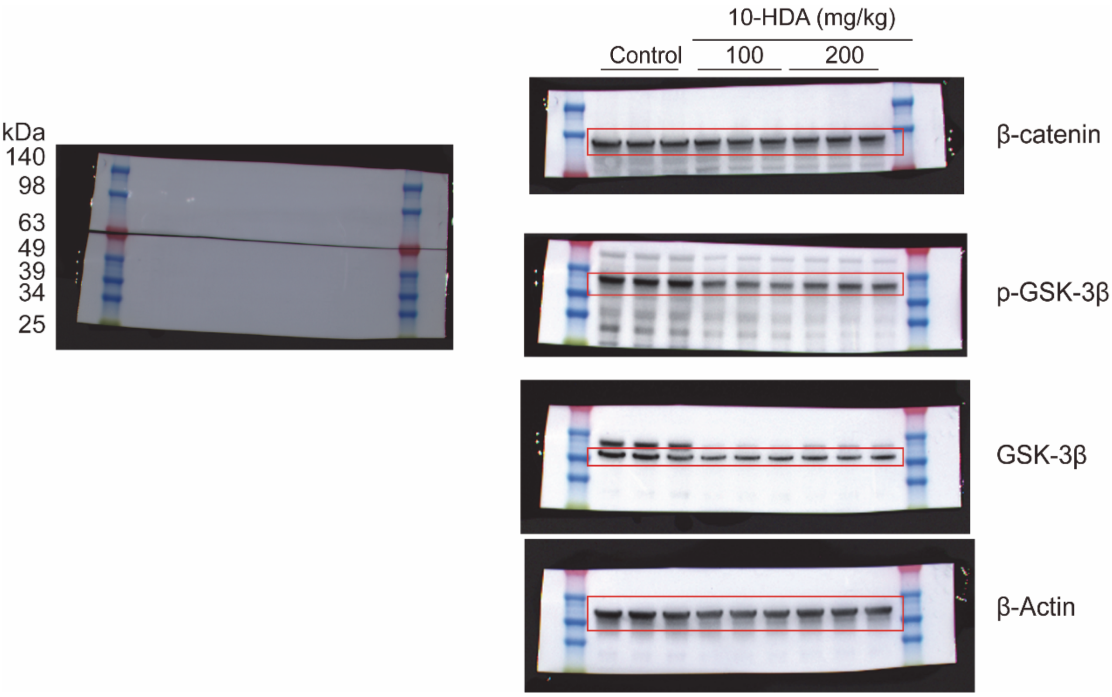

Source-DATA-Figure.6

Fig.6F: β-catenin

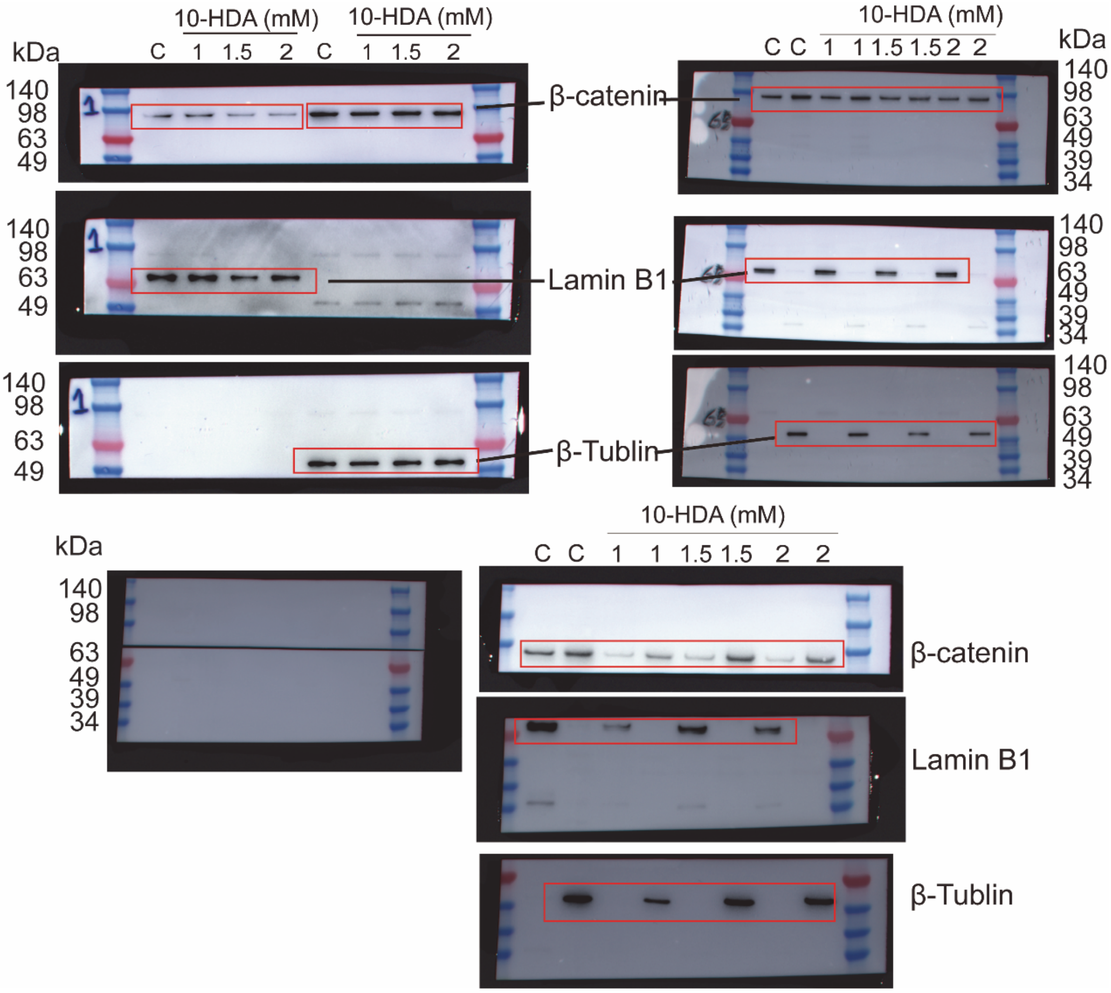

Supplement: Supplementary file 1 [file foods-15-01608-s001.zip › foods-4234613-supplementary/Supplementary Files/Supplementary Files/Figure S1.pdf]
