# Supplementary material for: 10-Hydroxy-2-decenoic Acid Suppresses Colorectal Cancer Progression by Inhibiting Wnt/β-Catenin Signaling and Promoting Apoptosis
Source: Foods. 2026 May 6;15(9):1608. doi: 10.3390/foods15091608 (PMC13163642; doi:10.3390/foods15091608)
Supplement: Supplementary file 1 [file foods-15-01608-s001.zip › foods-4234613-supplementary/Supplementary Files/Supplementary Files/Table S1.pdf]

Table S1. Primers used for qPCR

Gene	Forward (5'-3')	Reverse (5'-3')
ANO1	GAAGCGGAAACAGATGCGACTC	CTGGCTTCGTATTCAGCTCTAGG
MKI67	ACGCCTGGTTACTATCAAAAGG	CAGACCCATTTACTTGTGTTGGA
LTBP1	CTGACGGCCACGAACTTCC	GCACTGACATTTGTCCCTTGA
FGF19	TGCACAGCGTGCGGTACCTCT	CGGTACACATTGTAGCCATCTGG
NECTIN4	GCATCTACGTCTGCCATGTCAG	CTGACACTAGGTCCACCTGCTT
PLD1	TCCTGAAACGCCCAGTGGTTGA	ATGCCAAGAGCGAGTTCCACCT
SKA1	CGTTCCTTCCCATTTGCCTCAAG	GGAGGCTTCTTTACGGGTTCAG
KRT6B	GCCAAGGCAGACACTCTTACAG	TGGATAGCACCACGGATGTGTC
BCL-2	GGTGGGGTCATGTGTGTGG	CGGTTCAGGTACTCAGTCATCC
BAX	TCAGGATGCGTCCACCAAGAAG	TGTGTCCACGGCGGCAATCATC
BNIP2	TCCTAGTGATGGCTCTGTATTGT	ACTATTCTCTGACGGTGTGTCT
Caspase 3	GGAAGCGAATCAATGGACTCTGG	GCATCGACATCTGTACCAGACC
WNT11	CTGTGAAGGACTCGGAACTCGT	AGCTGTCGCTTCCGTTGGATGT
RNF43	GGTTACATCAGCATCGGACTTGC	ATGCTGGCGAATGAGGTGGAGT
β-Catenin	CACAAGCAGAGTGCTGAAGGTG	GATTCCTGAGAGTCCAAAGACAG
MMP7	TCGGAGGAGATGCTCACTTCGA	GGATCAGAGGAATGTCCCATACC
GAPDH	GTCTCCTCTGACTTCAACAGCG	ACCACCCTGTTGCTGTAGCCAA
